# Resection of the Intraductal Growth Type of Intrahepatic Cholangiocarcinoma Following a Long-Term Observation: a Rare Case Report

**DOI:** 10.1007/s12029-015-9768-2

**Published:** 2015-10-12

**Authors:** Takashi Okumura, Takao Ide, Atsushi Miyoshi, Kenji Kitahara, Hirokazu Noshiro

**Affiliations:** Department of Surgery, Saga University Faculty of Medicine, 5-1-1 Nabeshima, Saga, 849-8501 Japan

## Introduction

The incidence of intrahepatic cholangiocarcinoma (ICC) is reported to be 4.4 % of all primary liver tumors; it is the second most common type after hepatocellular carcinoma, and the 5-year survival rates are 12.2 % for non-surgical cases and 31.3 % for cases that were treated surgically [1]. However, the intraductal growth type of ICC shows different characteristics, with the tumor developing very slowly, providing a better prognosis [2]. We herein describe a case of ICC that was curatively resected after a 5-year observation. This case report is particularly interesting because it improves our general understanding of a subtype of ICC.

## Case Report

A 71-year-old male underwent distal gastrectomy for gastric cancer of T3N2M0, stage IIIA according to the AJCC/UICC pathological staging system [3] at another hospital in 2002. He had received periodic CT examinations every 6 months as surveillance after surgery. In June 2006, 4 years after the surgery, a mass lesion measuring less than 1 cm in diameter was found at Couinaud’s hepatic segment 5 (S5) in the liver. Thereafter, a CT examination was performed every 3 months to provide a more careful observation of the lesion. The shape of the tumor gradually changed from round to tubular, and the mass lesion temporarily decreased in size. During the first 3 years of the observation period, the size of the mass lesion remained relatively unchanged (Fig. 3). None of the serum tumor marker levels were elevated after the mass lesion was detected in the liver. The patient did not receive any chemotherapeutic agents during the observation period.

In August 2009, the patient visited our hospital due to new onset of epigastralgia. He was referred to our surgical department because of an abnormality found by an abdominal enhanced CT examination.

The patient weighed 53.0 kg, with a height of 145.6 cm, and showed no abnormalities in the physical examinations except for a surgical scar on the upper abdomen. His laboratory data on admission showed a slightly low hemoglobin (Hb) level of 12.9 g/dl and slightly elevated alkaline phosphatase (ALP) level of 387 U/ml; however, the other data were nearly within the normal ranges. The levels of tumor markers such as carcinoembryonic antigen (CEA), carbohydrate antigen (CA) 19-9, α-fetoprotein (AFP), and protein induced by vitamin K absence or antagonist (PIVKA)II were within the normal limits.

**Fig. 1:**
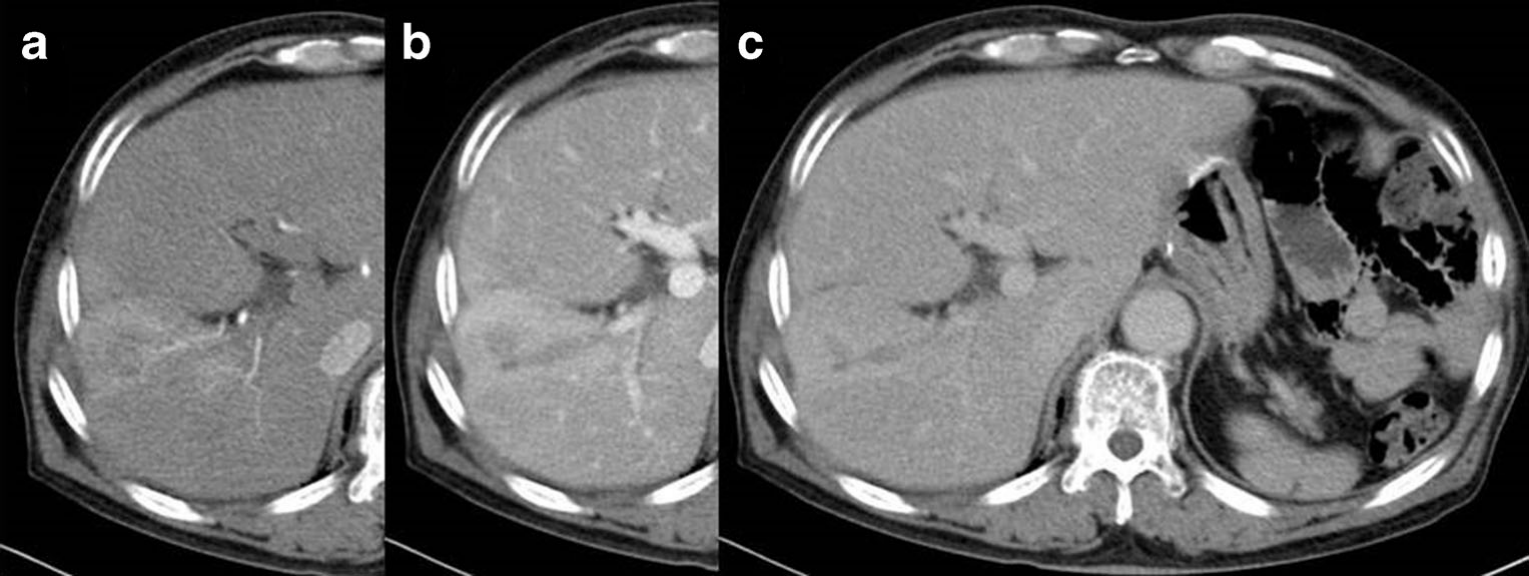
Abdominal contrast CT images of the liver tumor at the initial visit to our hospital in August 2009. **a** Arterial phase. **b** Portal phase. **c** Equilibrium phase

**Fig. 2:**
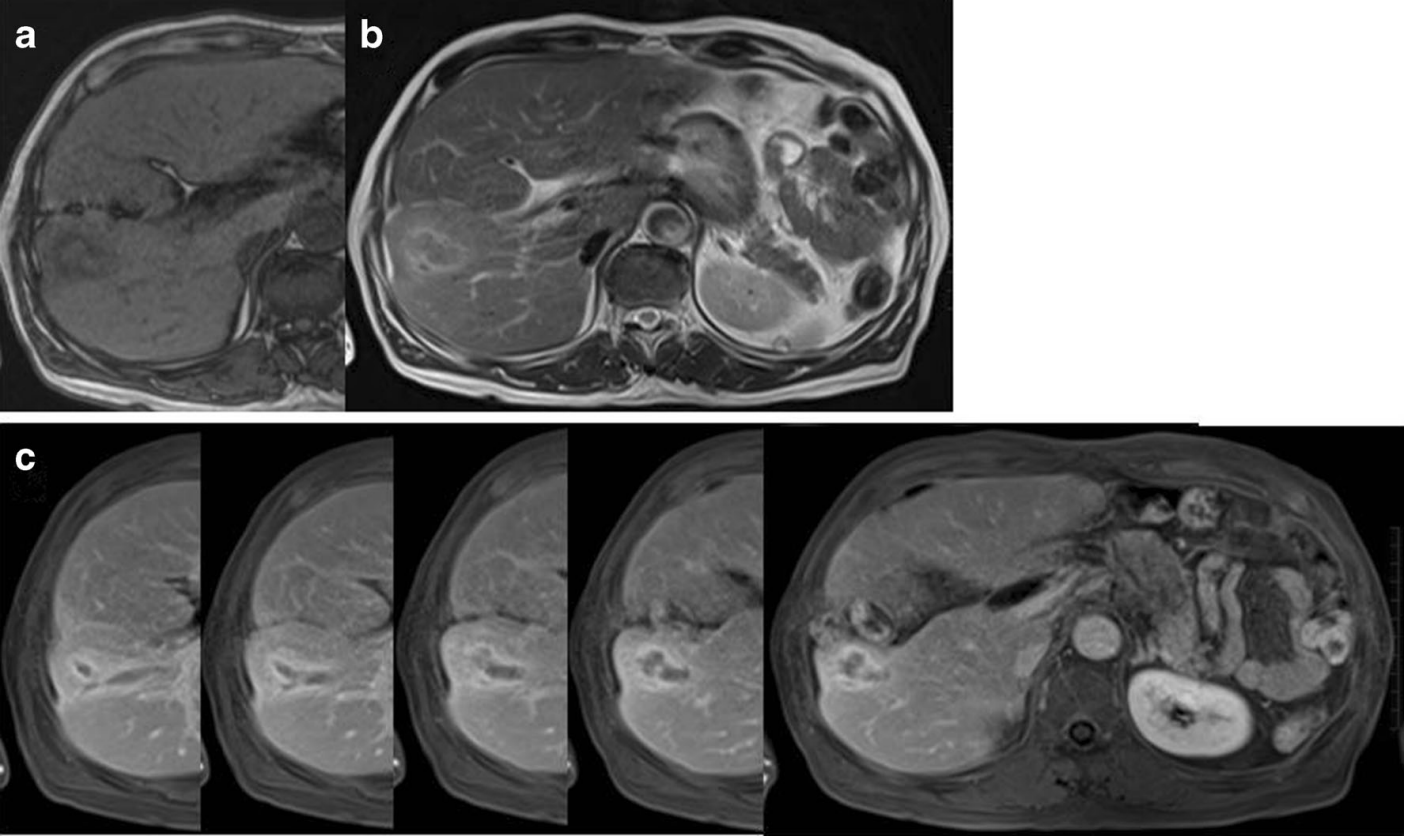
Abdominal contrast MRI images of the liver tumor at the initial visit to our hospital in August 2009. **a** T1W1. **b** T2W2. **c** Dynamic study

**Fig. 3:**
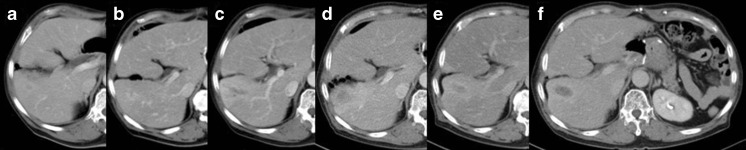
The changes in the CT images during the 5 years of observation. **a** June 2006. **b** May 2007. **c** July 2008. **d** Aug 2009. **e** Oct 2010. **f** July 2011

Abdominal contrast CT scans taken upon admission showed a tubular mass lesion along the Glisson’s sheath at S5 as a low density area. The border of the mass lesion was unclear and it was enhanced gradually from the early to late phases. The normal part of the liver surrounding the mass lesion was enhanced by the A-P shunt, and slight atrophy of the right lobe of the liver was seen (Fig. 1). Abdominal contrast MRI also showed the tubular lesion at S5, which appeared to be in the dilated bile ducts. The intensity of the center of the mass lesion was slightly high in T1W1 and equivalent in T2W1, and the edge of the mass lesion was low in T1W1 and slightly high in T2W1 (Fig. 2). According to the above findings, we considered that the mass lesion in the liver was likely to be a benign tumor, such as an inflammatory pseudotumor or a tumor formed due to some injury sustained during the previous operation, rather than primary or metastatic liver cancer. We advised the patient to undergo a needle biopsy or surgical resection to confirm the pathological diagnosis; however, he declined to undergo such an invasive examination. Therefore, careful observation was continued as had been performed at the previous hospital. However, after 2 years had passed, the low density mass lesion gradually increased in size. In July 2011, the size of the mass lesion reached 3 cm, and an atrophic change of the right lobe was observed (Fig. 3).

**Fig. 4:**
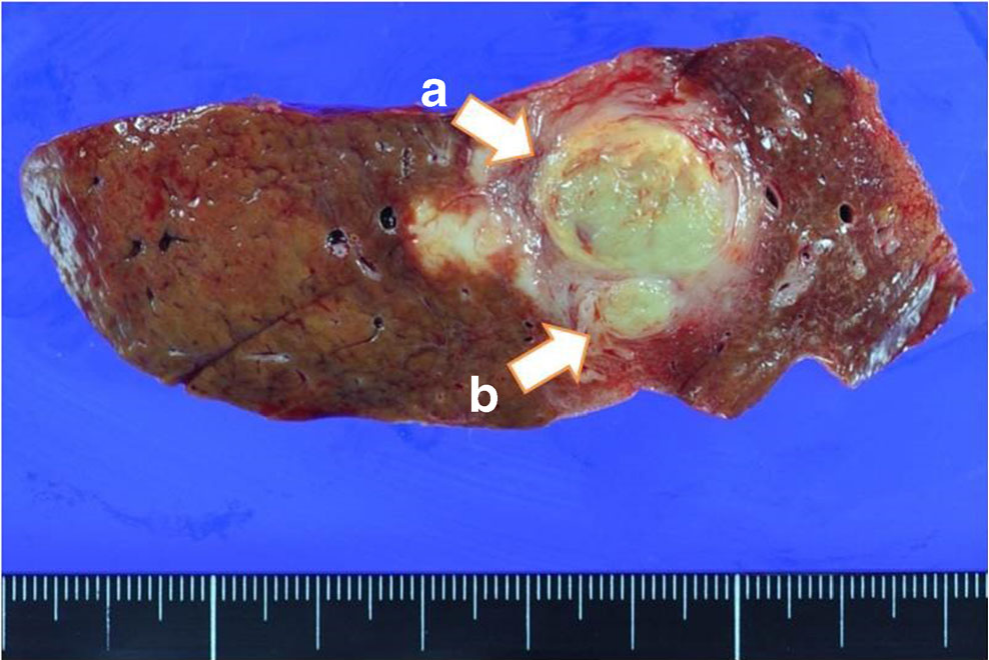
The macroscopic findings of the surgical specimen. **a** A 3 × 2 cm solid mass invading to the liver parenchyma and Glisson’s sheath. **b** The intraductal growth of the lesions in the B5 bile duct

**Fig. 5:**
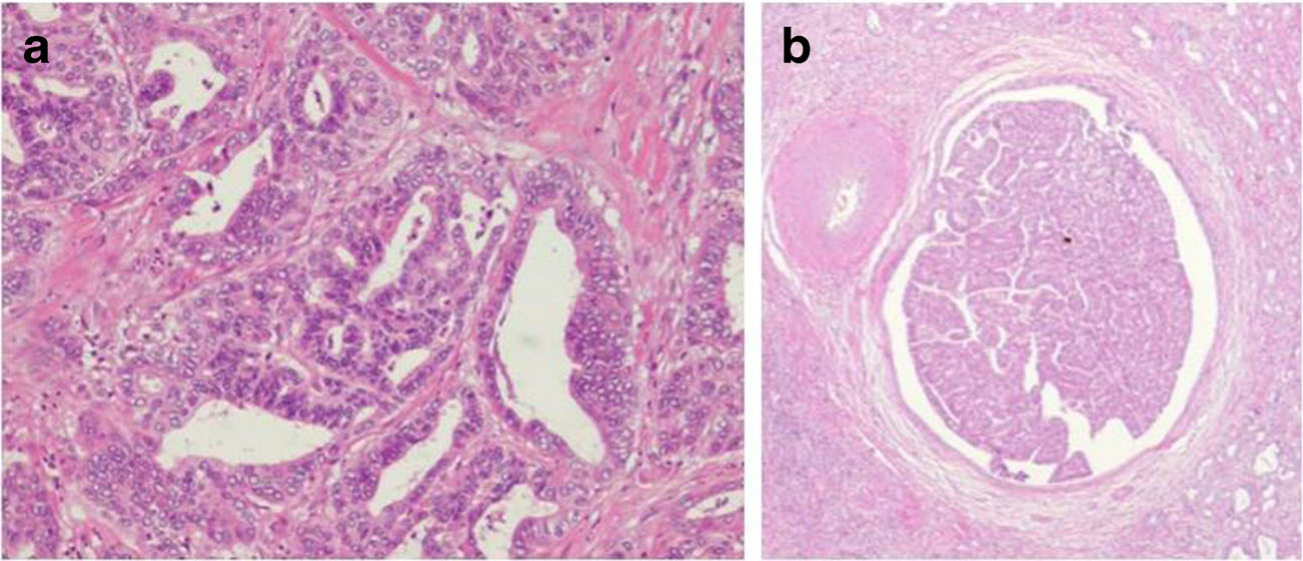
The microscopic findings of the surgical specimen. **a** The mass lesion was moderately differentiated adenocarcinoma. H&E, ×100. **b** The bile duct was filled with the intraductal growth lesion. H&E, ×40

After obtaining informed consent from the patient, we performed right hepatic lobectomy under the preoperative diagnosis of suspected ICC. The pathological examination of the excised specimen revealed that the mass lesion was cholangiocellular carcinoma (moderately differentiated adenocarcinoma) at S5/6 of the liver, and the tumor cells had spread along the bile duct of B5 similar to a tumor thrombus (Figs. 4 and 5). The final diagnosis according to the General Rules for the Clinical and Pathological Study of Primary Liver Cancer [4] was as follows: intrahepatic cholangiocarcinoma, mass-forming plus intraductal growth type, moderately differentiated adenocarcinoma; H2, St-AP, 3.0 cm, ig, fc(−), sf(−), s0, nX, vp1, vv0, b2, IM0, SM(−), NL.

The patient had an uneventful postoperative course and was discharged on the 17th day after the operation. We advised the patient to undergo adjuvant chemotherapy; however, he refused. The patient has shown no signs of recurrence 24 months after liver surgery.

## Discussion

We herein described a case of ICC that was curatively resected after 5 years of observation. To the best of our knowledge, there is no previous report of curatively resected ICC after such a long-term strict observation.

According to the World Health Organization (WHO) classification of tumors, ICC is defined as an intrahepatic malignant tumor composed of cells resembling those of the bile duct. ICC arises from any portion of the intrahepatic bile duct epithelium, such as the intrahepatic large bile duct or intrahepatic small bile duct. Cholangiocarcinoma arising from the right and left main hepatic ducts or their junction is referred to as hilar cholangiocarcinoma [5]. According to the General Rules for the Clinical and Pathological Study of Primary Liver Cancer, ICC is classified into three types: the mass-forming (MF) type, periductal infiltrating (PI) type, and intraductal growth (IG) type [4]. This classification is also mentioned in the WHO Classification of Tumors [5] and is now used all over the world. Although this classification is based on macroscopic findings, it is also related to the pathological features and prognosis of the tumors. Among the three types, the IG type spreads only in the bile duct lumen, with less lymph node and liver metastasis, thus leading to a better prognosis [2].

According to progressive features, two distinct neoplastic lesions have been identified thus far [6]. One is a flat or micropapillary growth of atypical biliary epithelium, referred to as biliary intraepithelial neoplasia (BilIN), which is known as one of the precursor lesions of ICC according to the WHO’s Classification of Tumors [5]. The other is an intraductal papillary neoplasm of the bile duct (IPN-B) with malignant potential, which is histologically characterized by the prominent papillary growth of atypical biliary epithelium with distinct fibrovascular cores and frequent mucin overproduction [7, 8]. IPN-B is considered to be a counterpart of the intraductal mucinous neoplasm of the pancreas (IPMN-P) because they have similar features, such as a macroscopic growth pattern of intraductal papillary proliferation [9], potential progression to tubular adenocarcinoma and mucinous carcinoma [9], increased expression of gastrointestinal metaplasia [10], and a favorable prognosis after surgical resection [11].

Although the present case did not have mucin production, the tumor did gradually increase in size. We speculated that the tumor in the B5 region might initially have been an intraductal neoplasm equivalent to BilIN or IPN-B. Subsequently, repeated blockage and healing of the peripheral bile duct might have caused the neoplasm to gain malignant potential gradually, finally contributing to its acquiring the ability to invade and form the mass lesion outside of the bile duct.

On the other hand, histological examinations of the present case revealed that the intraductal growth lesion demonstrated significant neutrophil invasion and tumor necrosis. These findings were consistent with the finding that the tumor temporarily decreased in size during the observation period. Although the IG type of ICC has a different biological behavior from the other types of ICC, a further understanding of the pathological and molecular biological features is required. Therefore, this case is particularly interesting because it contributes to understanding the IG type of ICC.

In the present case, we considered that surgical resection should have been performed in the earlier period, because the mass lesion increased in size, resulting in progressive atrophy of the right lobe of the liver. In addition, we must continue to pay attention to recurrent or new lesions in this case, because the tumor consisted of the IG component, as well as the MF component.
